# A screening assistance system for cervical cytology of squamous cell atypia based on a two‐step combined CNN algorithm with label smoothing

**DOI:** 10.1002/cam4.4460

**Published:** 2021-11-28

**Authors:** Yuta Nambu, Tasuku Mariya, Shota Shinkai, Mina Umemoto, Hiroko Asanuma, Ikuma Sato, Yoshihiko Hirohashi, Toshihiko Torigoe, Yuichi Fujino, Tsuyoshi Saito

**Affiliations:** ^1^ Department of Media Architecture Future University Hakodate Hakodate Japan; ^2^ Department of Obstetrics and Gynecology Sapporo Medical University School of Medicine Sapporo Japan; ^3^ Department of Pathology 1st Sapporo Medical University School of Medicine Sapporo Japan

**Keywords:** cervical cytology, deep learning, machine learning, ResNeSt, You Only Look Once (YOLO)

## Abstract

**Background:**

Although many cervical cytology diagnostic support systems have been developed, it is challenging to classify overlapping cell clusters with a variety of patterns in the same way that humans do. In this study, we developed a fast and accurate system for the detection and classification of atypical cell clusters by using a two‐step algorithm based on two different deep learning algorithms.

**Methods:**

We created 919 cell images from liquid‐based cervical cytological samples collected at Sapporo Medical University and annotated them based on the Bethesda system as a dataset for machine learning. Most of the images captured overlapping and crowded cells, and images were oversampled by digital processing. The detection system consists of two steps: (1) detection of atypical cells using You Only Look Once v4 (YOLOv4) and (2) classification of the detected cells using ResNeSt. A label smoothing algorithm was used for the dataset in the second classification step. This method annotates multiple correct classes from a single cell image with a smooth probability distribution.

**Results:**

The first step, cell detection by YOLOv4, was able to detect all atypical cells above ASC‐US without any observed false negatives. The detected cell images were then analyzed in the second step, cell classification by the ResNeSt algorithm, which exhibited average accuracy and F‐measure values of 90.5% and 70.5%, respectively. The oversampling of the training image and label smoothing algorithm contributed to the improvement of the system's accuracy.

**Conclusion:**

This system combines two deep learning algorithms to enable accurate detection and classification of cell clusters based on the Bethesda system, which has been difficult to achieve in the past. We will conduct further research and development of this system as a platform for augmented reality microscopes for cytological diagnosis.

## Graphical abstract

Through two different types of CNNs, we were able to exclusively detect and determine atypical cell clusters with high sensitivity. The deep learning approach was also extremely useful for cervical Pap Smears with diverse images.

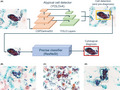

## 1 INTRODUCTION

The Cytoanalyzer^1^ was developed in the United States as the first automated diagnostic support system for cytology in 1957. This system classified cells into two classes based on nuclear size and density. However, this system detected clumps of blood cells, connections between tissues and mucosa, and overlapping epithelial cells as abnormalities and thus had a low accuracy of 36.6%.^1^ Thereafter, there were attempts to develop similar systems in the UK^2^ and Japan,^3^ but neither were put into clinical use. In the 1990s, the importance of obtaining specimens with minimized cell overlap for the establishment of automated screening systems was emphasized, and the ThinPrep^4^ and SurePath^5^ systems were developed. These systems use a method called liquid‐based cytology (LBC). The AutoPap system (BD Tripath Imaging), an automatic cytological diagnostic system that received FDA approval for smear use in 1998, was approved for use with SurePath slides in 2002.^6^ In 2003, the FDA approved TIS (Hologic) as the primary screener for ThinPrep Pap slides, and in 2008, the FDA approved the FocalPoint Guided Screening (GS) imaging system. However, LBC performed so well in clinical trials against smears that it also found a market apart from automated screening.^6^

In LBC, unlike conventional smears, cytology slides have a clean and uniform background, which is essential for the construction of an automatic cytological diagnostic system. Since the development of LBC, research on automated cytological classification has progressed dramatically. Two‐class classification (normal or cancer cells) for single‐cell images is the easiest and most accurate classification approach reported in the literature. A classification method based on estimating the width and center of gravity of cytoplasm and inputting values using support vector machine (SVM) methods has reached 98.61% accuracy in classification.^7^ A previous study using the AlexNet convolutional neural network (CNN) achieved classification with 99.3% accuracy, and the same study classified samples into seven classes with 93.75% accuracy using a unique CNN architecture for the Herlev dataset.^8^, ^9^ The Herlev dataset is a Pap‐smear dataset of cases from the University Hospital (Denmark) and consists of single cell images with nuclear positions. The C‐means method implementations have exhibited 98.88% accuracy^10^ and 99.6% accuracy in an algorithm using a unique CNN architecture.^11^ Other studies have reported accuracies of approximately 99%.^12^, ^13^, ^14^ Furthermore, multiclass classification for single‐cell images has also been reported to be highly accurate. A classification system constructed by inputting feature values, such as the ratio of nucleus and cytoplasm areas, into an SVM achieved 93.7% accuracy in seven‐class classification for single‐cell images.^15^ Other SVM‐based methods have successfully classified samples as negative for intraepithelial lesion or malignancy (NILM) or low‐ or high‐grade squamous intraepithelial lesions (LSIL or HSIL, respectively) with 88.88% accuracy.^16^ This indicates that it is possible to classify single‐cell images with high accuracy using existing techniques.

However, even though LBC specimens are ideally processed, the existence of crowded and overlapping cells in cell images is inevitable. Therefore, there is a serious need for an algorithm that can classify overlapping cell clusters accurately to enable the implementation of a human‐like automatic diagnostic system. As a classification method for multiple‐cell images, most two‐class classification methods obtain high accuracy by separating multiple‐cell images into single‐cell images. Zhang et al. simply divided multiple‐cell images into grids, automatically determined whether each grid contained background or not, and then extracted non‐background images.^17^ They used the features obtained from the wavelet transform of the non‐background images as input to classify normal and abnormal cells using an SVM method. They succeeded in classifying samples into two classes with an accuracy of 86–99% based on the true positive rate.^17^ In another study, Zhao et al. divided multiple cell images into a grid in the same way and classified each grid image into two classes with an accuracy of 98.98% using SVM.^18^ However, they did not try to classify the grade of atypia, and cell clusters were excluded from the determination of atypia before classification. Although SVM‐based systems are considered to be limited in detailed classification, these results indicate that two‐class classification for multiple‐cell images can also be achieved with high accuracy. However, two‐class classification alone is insufficient for applications in actual clinical practice, and multi‐class classification is thus required. However, the accuracy of multi‐class classification for multiple‐cell images is still insufficient. For example, Gautam et al. proposed a method for segmenting the nuclear region of each single cell and inputted the obtained nuclear regions into AlexNet‐based CNNs.^8^ An accuracy of 78.25% was obtained in four‐class classifications of NILM, LSIL, HSIL, and SCC. In recent years, general object detection methods have been proposed for detecting abnormal cells from multiple‐cell images and classifying them into multiple cytological classes based on CNN. For example, Xu et al. used Faster R‐CNN to classify samples into five classes,^19^ namely atypical squamous cells of undetermined significance (ASC‐US), LSIL, HSIL, endocervical cells (EC), and mesenchymal stromal cells (MSC), achieving 87.52% precision (i.e., positive predictive value) and 44.46% recall (i.e., sensitivity) on average. The low recall value indicates that atypical cells were often missed in their system. Because cervical cytology tests are generally performed as a cancer screening method, a high recall value is the most important performance requirement of the system. Therefore, the diagnostic accuracy of previous studies is insufficient for clinical use.

In the present study, we developed an automated method for detecting and classifying squamous atypical cells from overlapping and crowded multiple‐cell images with high diagnostic accuracy using a two‐step detection and classification system. The algorithm consisted of a first‐step location detector of atypical cells using You Only Look Once v4 (YOLOv4)^20^ and a second‐step detailed classifier using ResNeSt.^21^ The developed algorithm enables highly sensitive and near real‐time diagnosis support of cervical cytology.

## 2 MATERIALS AND METHODS

### 2.1 Ethics approval and consent to participate

Informed consent was obtained via opt‐out on the website of Sapporo Medical University. Those who opted out of the study were excluded. This investigation of the deep learning‐based cytological diagnosis assistance system was approved by the Ethics Committee of Sapporo Medical University.

### 2.2 Image preparation and processing

For machine learning with YOLOv4 and ResNeSt, image datasets of cervical cytology samples were constructed. The cytological images of LBC specimens (Cellprep^®^, Roche Diagnostics, Basel Switzerland) were acquired using a Nikon ECLIPSE Ci upright microscope (Nikon) and DS‐Ri2 digital camera (16.25 megapixels; Nikon). The focus was manually adjusted, and the depth of focus was searched for to best fit the entire area of each cell cluster. A total of 919 cell clusters of cytological images were captured to be classified into six cervical cytological classes based on the Bethesda classification system (NILM, ASC‐US, LSIL, ASC‐H, HSIL, and squamous cell carcinoma [SCC]). This study was limited to cervical squamous atypia, and the Bethesda classifications of atypical glandular cells, such as AGC and Adenocarcinoma, were excluded from the scope of this study. The specimens were randomly selected from cases of Pap tests performed at Sapporo Medical University between 2015 and 2018. The distribution of the datasets is summarized in Table 1.

**TABLE 1:** Number of images used for machine learning for each CNN algorithm

Bethesda classification	Number of cases	YOLOv4 datasets				ResNeSt datasets			
		Training datasets	Post data augmentation	Validation datasets	Test datasets	Training datasets	Post data augmentation	Validation datasets	Test datasets
NILM	26	0	0	0	100	118	4248	30	26
ASC‐US	35	104	3744	22	22	121	4356	32	26
LSIL	33	123	4428	26	26	114	4104	29	25
ASC‐H	29	138	4968	29	29	131	4716	34	28
HSIL	24	119	4284	26	26	201	7236	51	44
SCC	14	91	3276	19	19	267	9612	68	58

**FIGURE 1:**
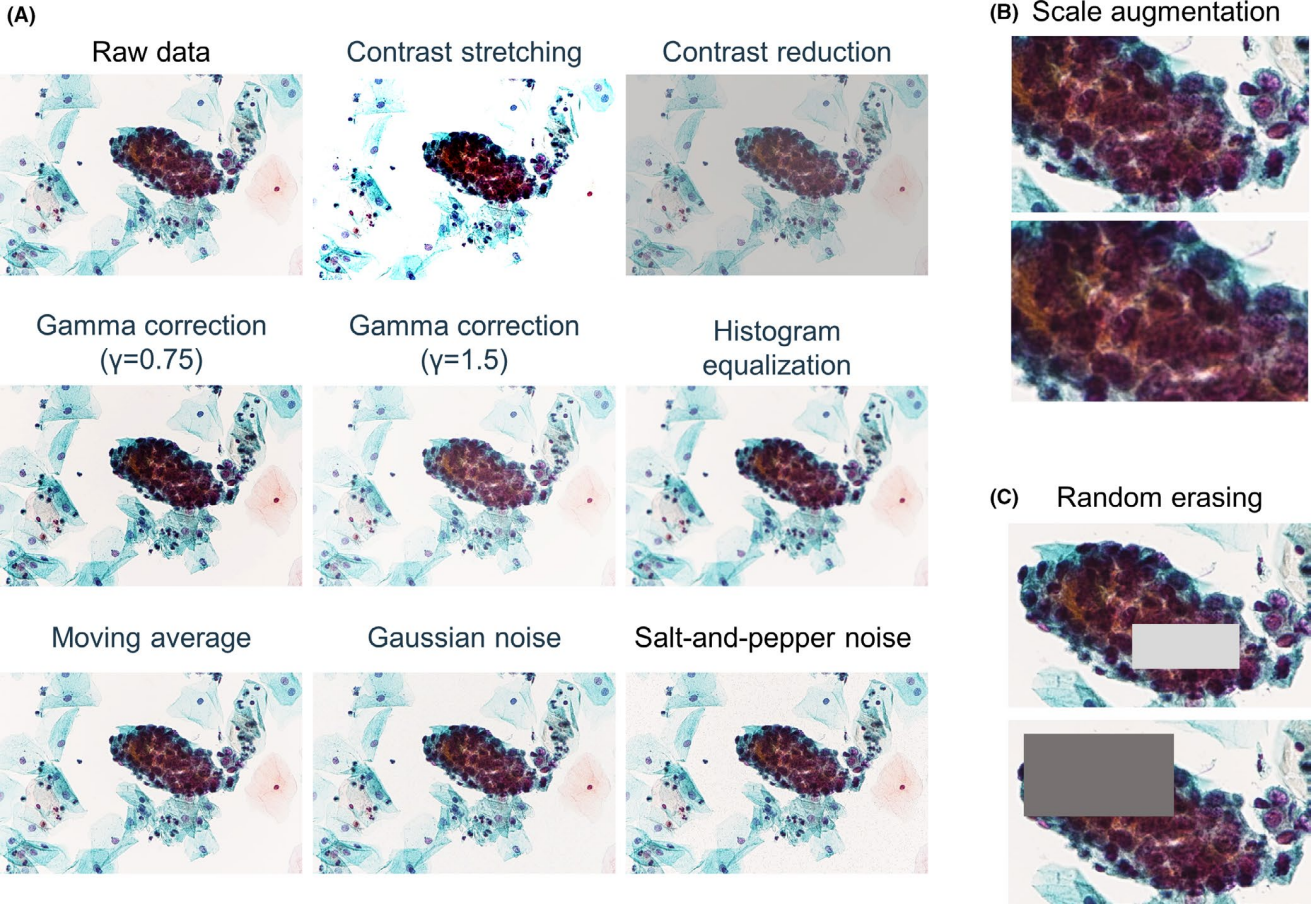
Oversampling methods applied to training data. (A) An example of data augmentation with nine patterns of filtering. The datasets were further increased by 36× by adding patterns to horizontal and vertical reverse images. (B, C) Scale Augmentation (B) and Random Erasing (C) applied for ResNeSt training

The magnification was 400×, and the size of the captured cell image was 1636 × 1088 pixels. Two specialists in cytology (one obstetrician and one pathologist) and one cytologist discussed and annotated the images of each Bethesda class. An estimated Bethesda classification was assigned to each cell group as an annotation. Of the annotated datasets, 70% were used as a training dataset, 15% as a validation dataset, and 15% as a test dataset. In applying the YOLOv4 algorithm, the NILM classification was included only in the test dataset because it is exclusively determined based on a sample result not being classified into any of the five atypical cell classes. YOLOv4 is based on a single image containing multiple cell clusters, whereas ResNeSt is based on the same image, though the number of datasets differs because they were constructed for each cell cluster. We oversampled the training dataset to improve the learning accuracy. Oversampling is the process of padding the amount of data to increase the learning accuracy by processing images with various filters when there are not enough images to serve as training data. First, both vertical and horizontal inversion as well as nine types of image filtering were performed (Figure 1A). These filters are intended to increase the robustness of the model by mimicking the light exposure and diverse specimen states across a variety of image capture environments. In addition, we used Scale Augmentation (Figure 1B)^22^ and Random Erasing (Figure 1C)^23^ together only for the ResNeSt training data. Scale Augmentation is performed by randomly enlarging the input image to a predetermined range of sizes and then randomly cropping out an image of the original size as the input image from that image. Random Erasing is a technique that conceals pixels in a portion of the input image. These two techniques have been shown to improve the accuracy of CNN‐based image classification models.

### 2.3 Two‐step diagnosis based on a combination of CNN algorithms

**FIGURE 2:**
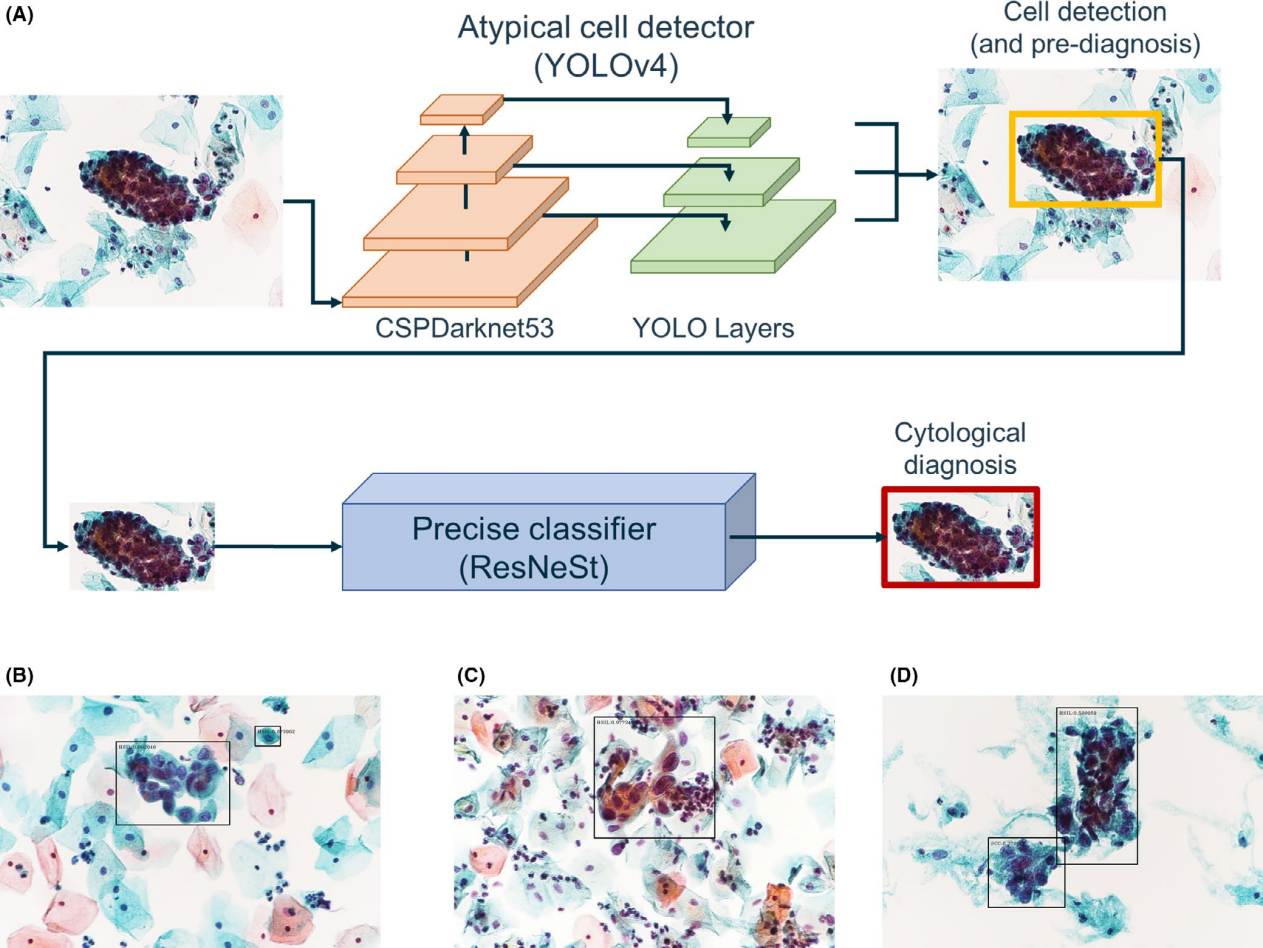
Algorithm of the two‐step CNN diagnostic system. (A) Framework of the two‐step cytological diagnosis by YOLOv4 and ResNeSt. B‐D. Images of atypical cells identified by the YOLOv4 algorithm. The atypical cell clusters are properly identified regardless of the background cell density

We used a computer with an accelerated computing unit (NVIDIA GeForce RTX 2070 super GPU) for machine learning calculation. Training, validation, and testing were performed using the constructed image dataset. The investigated system consists of two CNNs, YOLOv4^20^ and ResNeSt,^21^ corresponding to the detection step and classification step, respectively (Figure 2A). In this system, we decided to use individual learning of each CNN, YOLOv4 and ResNeSt, instead of end‐to‐end learning for efficiency of both memory usage and learning. If end‐to‐end learning had been used, YOLOv4 and ResNeSt would have needed to be loaded into memory at the same time. Then, the ResNeSt parameters would have needed to be frozen for YOLOv4 learning, while the YOLOv4 parameters would have needed to be frozen for ResNeSt learning. For the detection of atypical cells using YOLOv4, training was performed using the Darknet machine learning library.^20^ All hyperparameters in the training were set as recommended by the developers of YOLOv4. Clusters of atypical cells were detected within boundary boxes (Figure 2B–D). In the second step, the detailed classifier using ResNeSt was trained using the Tensorflow machine learning library. The parameters image size, number of channels, and learning rate were set to their default values, and the batch size was set to 16, which is the maximum value that can be loaded on the graphics processing unit (GPU) used in the study. The maximum number of iterations was set to 10,000 as recommended by the Darknet manual. During the iterations, the mean average precision (mAP) for the validation dataset was evaluated, and the model parameter obtaining the highest value before overfitting was saved. The training loss and validation mAP plots in this process are shown in Figure S1. Figure S1A shows that the decrease in loss slowed down and the accuracy of mAP did not improve from about 2300 batches in training without oversampling. Additionally, the same trend was observed in training with oversampling from about 6400 batches in Figure S1B, and this was determined to be the boundary for overfitting. In this algorithm, the model with oversampling is used because mAP increased from 49.3% without oversampling to 57.1% with oversampling. For ResNeSt, we used accuracy instead of mAP for the evaluation of the learning process (Figure S1C). Similarly, training was terminated when the training loss became flat and an increase in accuracy was no longer observed. We chose the model at 300 batches as the final model for ResNeSt.

Because the progression of cervical cancer from a healthy cervix is a continuous process, atypical cells at the boundary of Bethesda classifications are often visually similar. Therefore, when classifying crowded images of multiple cells, it is not suitable to make a single absolute classification. In this study, we used a “label smoothing” algorithm^24^ for the ResNeSt training data to achieve a smooth classification. The Cross Entropy function, used as the loss function of ResNeSt, is defined as

$$\text{Loss}=‐\sum_{i=1}^{c}y_{i}\text{log}\hat{y_{i}},$$

where *c* is the number of classes, $y\in R^{c}$ is the one‐hot‐encoded label data, and $\hat{y}\in R^{c}$ is the output vector of the model. This loss function allows the output of unreasonable predictions for classes that are not the correct answer class and does not consider the co‐occurrence between each class.

When considering a sample for which the correct answer is *k*∈[1,*c*],

$$\begin{array}{c} \text{Loss}=‐\sum_{i=1}^{c}y_{i}\text{log}\hat{y_{i}}\\ =‐\left(0\cdot \text{log}\hat{y_{1}}+0\cdot \text{log}\hat{y_{2}}+\cdots +1\cdot \text{log}\hat{y_{k}}+\cdots +0\cdot \text{log}\hat{y_{c}}\right)\\ =‐\text{log}\hat{y_{k}}. \end{array}$$

This means that only the prediction $\hat{y_{k}}$ for the correct answer class is evaluated independently. In general object detection, the co‐occurrence between objects is unknown, so this is not a problem. However, there is room for improvement in the case in which obvious co‐occurrence could be inferred, such as in this study, where HSIL and SCC are likely to co‐occur, but NILM and SCC are not. Therefore, we performed co‐occurrence–based learning by smoothing the correct label *y* with $y_{i}=n^{‐\left|k‐i\right|}$, where *n* is an arbitrary positive integer and a hyperparameter. From this definition, we can see that

$$\begin{array}{c} \text{Loss}=‐\sum_{i=1}^{c}y_{i}\text{log}\hat{y_{i}}=‐\left(n^{‐\left|k‐1\right|}\cdot \text{log}\hat{y_{1}}+n^{‐\left|k‐2\right|}\cdot \text{log}\hat{y_{2}}+\cdots +n^{‐\left|k‐k\right|}\cdot \text{log}\hat{y_{k}}+\cdots +n^{‐\left|k‐c\right|}\cdot \text{log}\hat{y_{c}}\right). \end{array}$$

and all the terms are evaluated. Here, $n^{‐\left|k‐i\right|}$ behaves as a weight, with $n^{‐\left|k‐k\right|}=1$ for the correct class; the further the class is from the index of the correct class, the smaller the weight. This makes it easier for the output probabilities of neighboring classes to co‐occur. This label smoothing assumes that the correct class is assigned in the following order: NILM, ASC‐US, LSIL, ASC‐H, HSIL, and SCC. This approach was chosen based on the definition of the Bethesda classification system, such that classes with similar progression levels are visually similar. After training the algorithm, the contribution of each oversampling method to the improvement in diagnostic ability was checked, and then, the model that showed the highest F‐measure in the validation was used for testing. As system performance indicators, we used the following statistically calculated parameters: accuracy = (*TP* + *TN*)/(*TP* + FP + *FN* + TN); precision = TP/(*TP* + FP); recall = TP/(*TP* + FN); and F‐measure = 2 * precision * recall/(precision + recall). In the equations used for calculating these parameters, TN, TP, FN, and FP represent the numbers of true‐negative, true‐positive, false‐negative, and false‐positive images, respectively. Recall is synonymous with sensitivity, and precision is the positive predictive value.

## 3 RESULTS

Table 2 summarizes the results of the system performance evaluation of each step of YOLOv4‐based cell detection and ResNeSt‐based classification.

**TABLE 2:** System performance of each step of CNN‐based cell classification

Bethesda classification	YOLOv4 performance (first step)				ResNeSt performance (second step)			
	Accuracy	Precision	Recall	F‐measure	Accuracy	Precision	Recall	F‐measure
NILM	77.0%	100.0%	49.0%	65.8%	98.0%	92.3%	92.3%	92.3%
ASC‐US	76.6%	27.9%	86.4%	42.2%	87.2%	60.0%	41.4%	49.0%
LSIL	90.1%	64.3%	34.6%	45.0%	92.0%	76.2%	59.3%	66.7%
ASC‐H	89.6%	75.0%	31.0%	43.9%	92.1%	66.7%	90.3%	76.7%
HSIL	89.2%	52.3%	88.5%	65.7%	85.5%	57.9%	61.1%	59.5%
SCC	91.9%	51.4%	94.7%	66.7%	88.4%	77.6%	80.4%	78.9%
Average	85.7%	54.2%	67.0%	54.9%	90.5%	71.8%	70.8%	70.5%

### 3.1 Atypical cell detection by the YOLOv4 algorithm

For NILM, ASC‐US, HSIL, and SCC, recall was higher than precision, whereas for LSIL and ASC‐H, recall was lower than precision. Because there is a trade‐off between precision and recall, a comparison among these six classes should be based on the F‐measure. The F‐measure of NILM, HSIL, and SCC detection was about 65%, while the F‐measure of ASC‐US, LSIL, and ASC‐H was approximately 40%. The average detection speed was 47.8 ms per image. The average recall, which is an important measure of performance in the first step of atypical cell screening, was 72.5%. Although this average recall value seemed to be low, this may not be the case when considering the sensitivity of detection of all atypical cells. Table 3 shows the confusion matrix of the results.

**TABLE 3:** Confusion matrix of atypical cell detection in each step

		Predicted class in YOLOv4 (first step)						Predicted class in ResNeSt (second step)					
		NILM	ASC‐US	LSIL	ASC‐H	HSIL	SCC	NILM	ASC‐US	LSIL	ASC‐H	HSIL	SCC
True class	NILM	49	13	5	1	15	17	24	1	0	0	0	1
	ASC‐US	0	19	0	1	2	0	0	12	4	6	4	3
	LSIL	0	16	9	0	1	0	2	4	16	3	1	1
	ASC‐H	0	18	0	9	2	0	0	1	0	28	2	0
	HSIL	0	2	0	1	23	0	0	2	1	3	22	8
	SCC	0	0	0	0	1	18	0	0	0	2	9	45

YOLOv4 had a particularly difficult time with the classification of ASC‐US, exhibiting a precision of only 27.9%. This result is elucidated by inspection of the confusion matrix because many of the other classes were classified as ASC‐US. However, the positive predictive rates for HSIL and SCC were extremely high in the first step conducted with YOLOv4. In addition, there were no cases of atypical cells over ASC‐US that were underestimated as NILM. Therefore, when the cutoff was set as atypical cells above ASC‐US, the recall of the algorithm was 100%. Therefore, we consider that the system exhibits the necessary performance to function as a detector of atypical cells.

### 3.2 Precise classification by the ResNeSt algorithm

The second step, classification using the ResNeSt algorithm, was performed for the atypical cells detected in the first step. In the second step, we examined the contribution of each oversampling method on the validation results. Overall, the highest diagnostic performance was obtained when all oversampling methods and label smoothing were used. Compared with the model without oversampling (average F‐measure, 44.0 ± 3.3%), a combination of oversampling methods improved the average F‐measure by 10.0%. Furthermore, a combination of label smoothing improved the F‐measure by 7.1%, and the average F‐measure reached 61.1 ± 3.6% in the validation phase. Following this result, we tested the model trained with all oversampling methods; the results are summarized on the right side of Table 2, and the corresponding confusion matrix is shown in Table 3.

NILM showed the highest F‐measure of 92.3%, followed by SCC, with 78.9%. Because the sensitivity for atypical cells of the YOLOv4 was tuned higher to avoid missing atypical classes, there were many cases for which NILM was classified to be atypical. Therefore, the highest F‐measure for NILM was essential for the classification step of ResNeSt. The average of F‐measures for all classes was 70.5%, showing a marked improvement in diagnostic performance compared with the first step, YOLOv4. The differences between precision and recall were small for NILM and SCC and larger for ASC‐US, LSIL, and ASC‐H. The average classification speed was 77.6 ms per image in the developer's environment for the second step. In other words, a total of 122.2 ms per image was required for this two‐step cell diagnosis algorithm. Assuming that the system is applied to a whole slide image (WSI) that has already been captured, we calculated the time required. If the WSI of a slide is captured at 400× magnification using Nanozoomer (Hamamatsu Photonics K.K.), the total number of images is 224 given the input image size assumed in this method, which means that the method can be executed in 27.4 s. However, we expect that this result can easily be improved by using a faster GPU.

## 4 DISCUSSION

In developing our system, we placed the greatest emphasis on the accurate determination of multiple crowded cells. Previous assessments of classification algorithms have often used the Herlev dataset^9^ as a unified dataset for method comparison. One feature of this dataset is that the images are well suited for mechanical classification because they have been carefully captured and sorted and adjusted in order to reduce noise. However, comparisons of classification algorithms for single cells based on the Herlev dataset are not applicable to real clinical specimens. This is because not only are actual specimens of cervical cytology often captured as multi‐cell images but also background noise cannot be completely removed, even by LBC processing. To solve these problems, we used a combination of two CNN algorithms in this study, namely, YOLOv4^20^ and ResNeSt.^21^ We selected YOLOv4 as the detector for several key reasons. First, object detection methods based on deep learning can directly detect objects in an image, thus eliminating the need for preprocessing, such as noise removal and single‐cell segmentation. Additionally, among object detection methods, YOLOv4 achieves a good balance between detection speed and detection accuracy and is suitable as the first step in a multi‐step system. However, the most important reason is that the architecture of YOLOv4 is able to identify atypical cells among many other normal cells.

From the perspective of screening, it is important to be able to not only detect abnormal cells but also correctly recognize normal cells and ignore them in an algorithm. The architecture of YOLOv4 meets this performance requirement. YOLOv4 divides the entire target image into grids and then calculates the probability of belonging to one of the classification classes for each grid. In other words, YOLOv4 learns to set the probability to zero for regions in which there are no objects to be detected. Therefore, if the number of detected cells is zero for a model that has only been trained on atypical cells, the result is interpreted as the absence of atypical cells, and the cytological image is thus classified as NILM. This exclusive detection of normal cells eliminates the need to include normal cells in the training dataset. This is a great advantage because cervical cytology is highly variable, even for normal cells, and annotation of cells is a very time‐consuming process. This advantage is difficult to obtain with R‐CNNs,^25^ SPPnet,^26^ and Faster R‐CNNs,^27^ among other methods, in which the region extraction and classification algorithms are split. These machine learning methods do not have a way to reduce their sensitivity to normal cells.

We chose ResNeSt^21^ as the classifier for the second stage because of its high accuracy in analyzing the ImageNet dataset.^28^ There are six types of ResNeSt algorithms, which differ in their number of layers: ResNeSt14, ResNeSt26, ResNeSt50, ResNeSt101, ResNeSt200, and ResNeSt269. ImageNet is customarily used as a benchmark for image classification methods, and ResNeSt269 shows the highest accuracy in analyzing the ImageNet dataset (https://cv.gluon.ai/model_zoo/classification.html). However, as the number of layers increases, it has the disadvantages of slow detection speed and high memory usage. Therefore, we decided to use ResNeSt50 to achieve a balance among accuracy, detection speed, and machine memory usage. Our objective is to develop a system that can detect atypical cancer cells in real‐time and assist in the decision‐making process immediately, motivating our selection of an algorithm that balances the trade‐off between speed and accuracy. The average accuracy of 90.5% and F‐measure of 70.5% for our algorithm are relatively high compared with those for previously reported methods, but further improvement in diagnosis performance is needed for clinical use. In a systematic review, the sensitivity of Pap test screening by human cytoscreeners in detecting cervical dysplasia was 30% to 87%, and the specificity was 86% to 100%.^29^ Therefore, we consider it is necessary to aim for a value close to 100% accuracy in automated diagnostic algorithms.

To improve the accuracy, flexible assignment of data labels and increasing the amount of training data may be beneficial. Flexible assignment of correct labels means that label smoothing should be conducted to more closely resemble real images.^24^ In this study, we made the label smoothing criterion mountainous. A visualization of our label smoothing of $n^{‐\left|k‐i\right|}$ for *c* = 6 and *n* = 2, 3 is shown in Figure S2. For example, if HSIL is the most suspicious diagnosis (*k* = 4), we have implemented gentle labeling throughout such that the next highest probability is associated with the neighboring classes, ASC‐H (*i* = 3) and SCC (*i* = 5). This is only a hypothetical model, and the distribution of the Bethesda classification is not necessarily mountainous when considering the actual cell clusters. The label smoothing method alone contributed to a 7.1% F‐measure improvement, but further parameter adjustments specific to cell diagnosis may be necessary. It is possible that some improvement could be made in the implementation of label smoothing, such as considering the distribution of the cytodiagnostic classifications according to their approximation and frequency.

There are several limitations to our study. The first is the small size of the dataset used for training. Generally, more training datasets improve accuracy because they increase the coverage for the population in machine learning. The number of images analyzed in our study was about one‐hundredth of the number utilized in a previous study using Faster R‐CNN.^19^ This is because we created a high‐quality training dataset by taking pictures of cervical cell clusters with characteristic findings, determining the boundary region, and discussing the annotation with multiple experts. Thus, in this study, we used oversampling. Because oversampling can improve accuracy in machine learning, it is a method that is actively used in medical research, where training data tends to be scarce. In the sense that it can handle various imaging conditions, oversampling is a useful method because it is expected to improve robustness. However, oversampling can occasionally produce images that are very dissimilar from the original image, which may reduce accuracy. Consequently, there are some areas in which the achieved accuracy is not sufficient. In using this system for cervical screening, we are most concerned about false negatives. Although the first step by YOLOv4 successfully detects atypical cells, there are some classes that are underestimated by ResNeSt. It is important to note that there are cases of underestimation below ASC‐H classes, where surgical treatment is actively considered, such as HSIL and SCC (Table 3). An additional algorithm may need to be constructed to identify these cases individually if no improvement is achieved by increasing the number of dataset images. Furthermore, there are still difficulties in classifying borderline classes such as ASC‐US and ASC‐H. Clinically, the diagnosis of these classes is the basis for HPV testing or biopsy, and we need to consider how to classify them correctly. We assume that the difficulty is specific to each ASC class because we used almost the same amount of training data for all classes. Because these classes are not likely to consist of large cell clusters, it may be effective to use an ordinary machine learning algorithm established for single cells. In addition, atypical glandular cells were not included in the dataset analyzed. The main reason for this exclusion was that the number of cases of atypical glandular cells was small compared with atypical squamous cells, making it difficult to obtain an adequate dataset. Furthermore, because cell clusters of glandular cells have a more obvious three‐dimensional structure compared with squamous cell clusters, we are investigating other approaches that can classify three‐dimensional structures correctly.

As a second important limitation, the evaluation of learning accuracy may be insufficient. In this study, we captured images of the ideal field of view and annotated them with characteristic cell clusters. When evaluating these datasets, YOLOv4 can detect characteristic cell populations, and the classification by ResNeSt must ultimately produce the final accuracy. However, YOLOv4 may detect some extraneous objects as suspected atypical cells when the system is applied to suboptimal images. If end‐to‐end evaluation is to be done properly, all these potential false positives must be annotated. As mentioned above, we took great care in creating this dataset, and it was difficult to create additional datasets for further evaluation of suboptimal cytological images.

To pursue clinical use of this system, we propose continued research and development in the form of an augmented reality microscope (ARM). The ARM reported by Google in 2019^30^ enabled detection of breast cancer metastasis sites and prostate cancer in real time during speculum examination by using a system based on deep learning. We believe that our YOLO‐based assistance system with high detection rate and real‐time performance will be a good match for the ARM concept. We hope that its combined use with ARM in cytological screening will reduce the risk of missing atypical cells and reduce the workloads of cytoscreeners.

## 5 CONCLUSION

This two‐step algorithm was determined to provide highly sensitive classification results with clear potential to support near real‐time diagnosis in clinical cervical cytology. In future research, we will focus on the continued development of this system in conjunction with an augmented reality microscope for cytological screening assistance.

## CONFLICT OF INTEREST

T.M. received research funding from the Akiyama Life Science Foundation and the Noastec Research Foundation and reports no other conflict of interest. All the other authors report no conflicts of interest.

## AUTHOR CONTRIBUTIONS

Tasuku Mariya: Conceptualization, Methodology. Yuta Nambu: Visualization, Investigation, Software, Writing – Original draft preparation. Shota Shinkai, Hiroko Asanuma, and Mina Umemoto: Data curation. Yoshihiko Hirohashi, Toshihiko Torigoe, and Tsuyoshi Saito: Supervision. Ikuma Sato and Yuichi Fujino: Software, Validation.

## Supporting information

Fig S1

Fig S2

## Data Availability

The data that support the findings of this study are available from the corresponding author upon reasonable request.

## References

[1] Spencer CC , Bostrom RC . Performance of the cytoanalyzer in recent clinical trials. J Natl Cancer Inst. 1962;29:267‐276. 13915656

[2] Spriggs AI , Diamond RA , Meyer EW . Automated screening for cervical smears? Lancet. 1968;1(7538):359‐360. 10.1016/s0140-6736(68)90822-2 4170181

[3] Watanabe S . An automated apparatus for cancer prescreening: CYBEST. Comput Graph Image Process. 1974;3:350‐358.

[4] Hutchinson ML , Cassin CM , Ball HG 3rd . The efficacy of an automated preparation device for cervical cytology. Am J Clin Pathol. 1991;96(3):300‐305. 1877527 10.1093/ajcp/96.3.300

[5] Howell LP , Davis RL , Belk TI , Agdigos R , Lowe J . The AutoCyte preparation system for gynecologic cytology. Acta Cytol. 1998;42(1):171‐177. 9479336 10.1159/000331542

[6] Cibas ES , Ducatman BS . Cytology: Diagnostic Principles and Clinical Correlates. ELSEVIER; 2021. https://www.elsevier.com/books/cytology/cibas/978‐0‐323‐63636‐0

[7] Chen Y‐F , Huang P‐C , Lin K‐C , et al. Semi‐automatic segmentation and classification of Pap smear cells. IEEE J Biomed Health Inform. 2014;18(1):94‐108. 24403407 10.1109/JBHI.2013.2250984

[8] Gautam S , Harinarayan KK , Jith N , Sao AK , Bhavsar A , Natarajan A . Considerations for a PAP Smear Image Analysis System with CNN Features. 2018, June 01, 2018:[arXiv:1806.09025 p.]. Available from: https://ui.adsabs.harvard.edu/abs/2018arXiv180609025G

[9] Jantzen J , Norup J , Dounias G , Bjerregaard B . Pap‐smear Benchmark Data for Pattern Classification. Nature inspired Smart Information Systems: EU co‐ordination action. NiSIS; 2005:1‐9.

[10] William W , Ware A , Basaza‐Ejiri AH , Obungoloch J . A pap‐smear analysis tool (PAT) for detection of cervical cancer from pap‐smear images. Biomed Eng Online. 2019;18(1):16. 30755214 10.1186/s12938-019-0634-5 PMC6373062

[11] Nirmal Jith OU , Harinarayanan KK , Gautam S , Bhavsar A , DeepCerv SAK . DeepCerv: deep neural network for segmentation free robust cervical cell classification. Computational pathology and ophthalmic medical image analysis. Lecture Notes in Computer Science; Springer; 2018:86‐94. https://link.springer.com/chapter/10.1007%2F978‐3‐030‐00949‐6_11

[12] Chankong T , Theera‐Umpon N , Auephanwiriyakul S . Automatic cervical cell segmentation and classification in Pap smears. Comput Methods Programs Biomed. 2014;113(2):539‐556. 24433758 10.1016/j.cmpb.2013.12.012

[13] Zhang L , Le L , Nogues I , Summers RM , Liu S , Yao J . DeepPap: deep convolutional networks for cervical cell classification. IEEE J Biomed Health Inform. 2017;21(6):1633‐1643. 28541229 10.1109/JBHI.2017.2705583

[14] Guanglu Sun SL , Cao Y , Lang F . Cervical cancer diagnosis based on random forest. Int J Performability Eng. 2017;13(4):446‐457.

[15] Mariarputham EJ , Stephen A . Nominated texture based cervical cancer classification. Comput Math Methods Med. 2015;2015:586928. 25649913 10.1155/2015/586928 PMC4310228

[16] Bora K , Chowdhury M , Mahanta LB , Kundu MK , Das AK . Pap smear image classification using convolutional neural network. Proceedings of the Tenth Indian Conference on Computer Vision, Graphics and Image Processing ‐ ICVGIP ‘162016. p. 1‐8.

[17] Zhang J , Liu Y . Cervical cancer detection using SVM based feature screening. Lecture Notes in Computer Science. 2004;3217(1 PART 2):873‐880.

[18] Zhao M , Wu A , Song J , Sun X , Dong N . Automatic screening of cervical cells using block image processing. Biomed Eng Online. 2016;15:14. 26847685 10.1186/s12938-016-0131-z PMC4743397

[19] Meiquan X , Weixiu Z , Yanhua S , et al., Cervical Cytology Intelligent Diagnosis Based On Object Detection Technology. 2018. https://openreview.net/forum?id=r14Qkwsif

[20] Redmon J , Divvala S , Girshick R , Farhadi A . You Only Look Once: Unified, Real‐Time Object Detection. 2015 June 01, 2015:[arXiv:1506.02640 p.]. Available from: https://ui.adsabs.harvard.edu/abs/2015arXiv150602640R

[21] Zhang H , Wu C , Zhang Z , et al. ResNeSt: Split‐Attention Networks. 2020 April 01, 2020:[arXiv:2004.08955 p.]. Available from: https://ui.adsabs.harvard.edu/abs/2020arXiv200408955Z

[22] Shorten C , Khoshgoftaar TM . A survey on image data augmentation for deep learning. Journal of Big Data. 2019;6(1):60. 10.1186/s40537-021-00492-0 PMC8287113 34306963

[23] Zhong Z , Zheng L , Kang G , Li S , Yang Y . Random Erasing Data Augmentation. 2017 August 01, 2017:[arXiv:1708.04896 p.]. Available from: https://ui.adsabs.harvard.edu/abs/2017arXiv170804896Z

[24] Szegedy C , Vanhoucke V , Ioffe S , Shlens J , Wojna Z . Rethinking the Inception Architecture for Computer Vision. 2015 December 01, 2015:[arXiv:1512.00567 p.]. Available from: https://ui.adsabs.harvard.edu/abs/2015arXiv151200567S

[25] Girshick R , Donahue J , Darrell T , Malik J . Rich feature hierarchies for accurate object detection and semantic segmentation. 2013 November 01, 2013:[arXiv:1311.2524 p.]. Available from: https://ui.adsabs.harvard.edu/abs/2013arXiv1311.2524G

[26] He K , Zhang X , Ren S , Sun J . Spatial Pyramid Pooling in Deep Convolutional Networks for Visual Recognition. 2014 June 01, 2014:[arXiv:1406.4729 p.]. Available from: https://ui.adsabs.harvard.edu/abs/2014arXiv1406.4729H 10.1109/TPAMI.2015.2389824 26353135

[27] Ren S , He K , Girshick R , Sun J . Faster R‐CNN: Towards Real‐Time Object Detection with Region Proposal Networks. 2015 June 01, 2015:[arXiv:1506.01497 p.]. Available from: https://ui.adsabs.harvard.edu/abs/2015arXiv150601497R 10.1109/TPAMI.2016.2577031 27295650

[28] Krizhevsky A , Sutskever I , Hinton GE . ImageNet classification with deep convolutional neural networks. Proceedings of the 25th International Conference on Neural Information Processing Systems ‐ Volume 1. Curran Associates Inc.; 2012. 1097‐1105.

[29] Nanda K , McCrory DC , Myers ER , et al. Accuracy of the Papanicolaou test in screening for and follow‐up of cervical cytologic abnormalities: a systematic review. Ann Intern Med. 2000;132(10):810‐819. 10819705 10.7326/0003-4819-132-10-200005160-00009

[30] Chen P‐H , Gadepalli K , MacDonald R , et al. An augmented reality microscope with real‐time artificial intelligence integration for cancer diagnosis. Nat Med. 2019;25(9):1453‐1457. 31406351 10.1038/s41591-019-0539-7

