# Supplementary figures and images for: A screening assistance system for cervical cytology of squamous cell atypia based on a two‐step combined CNN algorithm with label smoothing
Source: Cancer Med. 2021 Nov 28;11(2):520–9. doi: 10.1002/cam4.4460 (PMC8729059; doi:10.1002/cam4.4460)

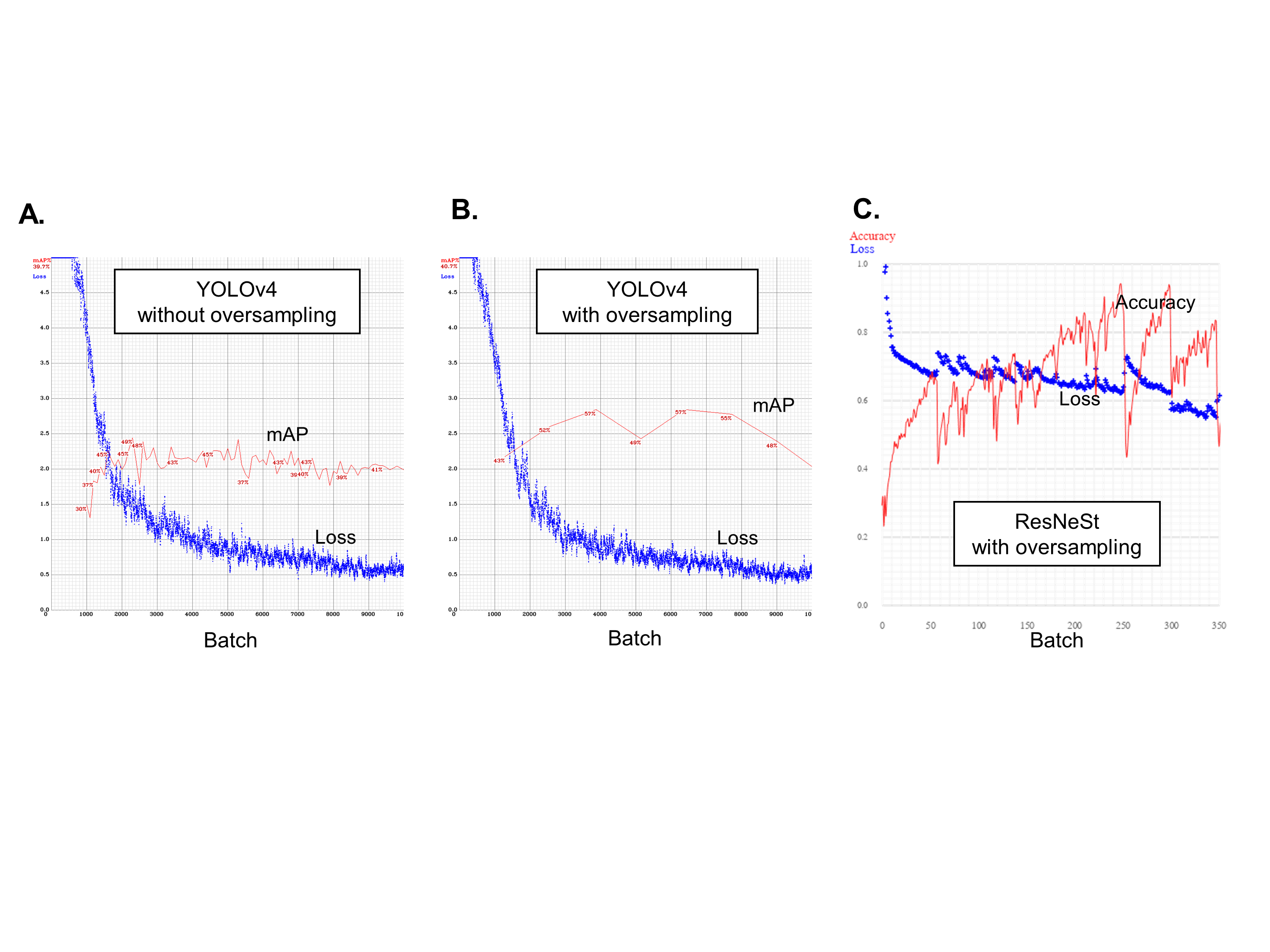

Supplement: Supplementary file 1 — Fig S1 [file CAM4-11-520-s001.tif]

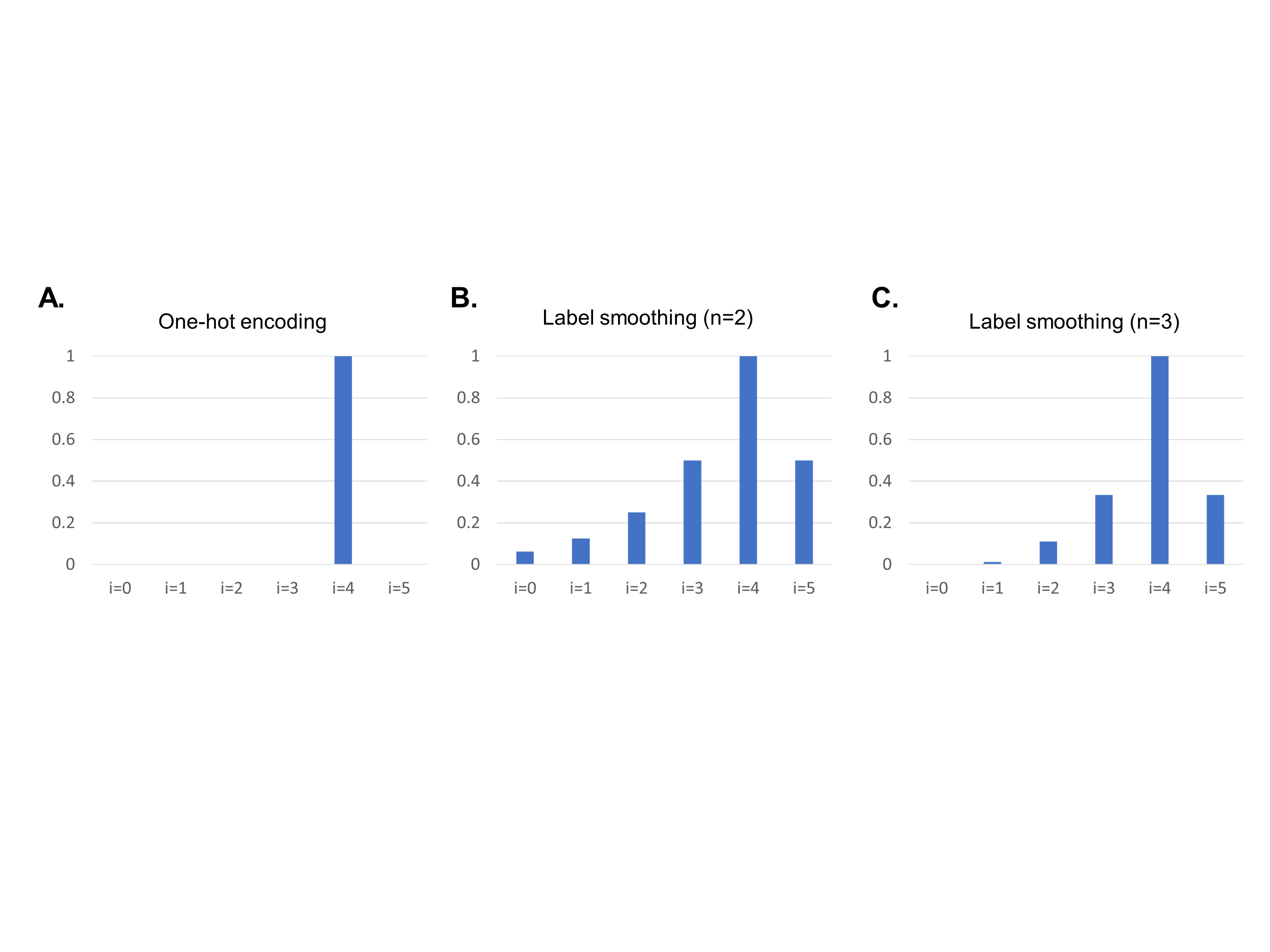

Supplement: Supplementary file 2 — Fig S2 [file CAM4-11-520-s002.tif]
